# Temporary Reactive Response of Axillary Lymph Nodes to COVID-19 Vaccination on ^18^F-rhPSMA-7.3 PET/CT in Patients with Prostate Cancer

**DOI:** 10.2967/jnumed.121.263758

**Published:** 2022-11

**Authors:** Susan Notohamiprodjo, Matthias Eiber, Christian Lohrmann, Wolfgang A. Weber

**Affiliations:** Department of Nuclear Medicine, Klinikum Rechts der Isar, Technical University of Munich, Munich, Germany

**Keywords:** COVID-19 vaccine, PSMA-ligand PET/CT, axillary lymphadenopathy, rhPSMA

## Abstract

Vaccine-associated lymphadenopathy (VAL) is a common finding on ^18^F-FDG PET/CT examinations after coronavirus disease 2019 (COVID-19) vaccination. However, data regarding VAL on ^18^F-rhPSMA-7.3-ligand PET are currently lacking. This study assesses the prevalence, temporal response to vaccination, and characteristics of VAL. **Methods:** Two hundred thirty-three consecutive vaccinated and 41 unvaccinated patients with confirmed prostate cancer who underwent ^18^F-rhPSMA-7.3 PET/CT were retrospectively analyzed. Size and uptake of the axillary lymph nodes were measured. Ratios of SUV_max_ of ipsilateral to contralateral axillary lymph node (SUVratio) were determined. The characteristics of SUVratio in respect to the duration of PSMA avidity in the axillary lymph node after COVID-19 vaccination was analyzed. **Results:** The prevalence of VAL on ^18^F-rhPSMA-7.3 PET was 45%. Up to a period of 8 wk after the last COVID-19 vaccination, SUVratio was positive (2.05 ± 0.17). Thereafter, SUVratio dropped significantly (1.35 ± 0.09) and approached the value of unvaccinated group (1.1 ± 0.2). SUVratio of metastatic axillary lymph nodes was very high (>11) and can be easily detected visually or semiquantitatively. In 3 patients, we observed suspected development and consecutively confirmed involving metastasis of axillary lymph node with SUVratio between 4.0 to 6.6. Correlation between SUVratio and lymph node size (*r* = 0.93, *P* < 0.0001) and lymph node size and duration after vaccine (*r* = −0.88, *P* < 0.0008) was found. **Conclusion:** Increased uptake of the PSMA ligand ^18^F-rhPSMA-7.3 by axillary lymph nodes is common after COVID-19 vaccination and can persist for 8 wk. This finding should be considered in the interpretation of ^18^F-rhPSMA-7.3 PET/CT examinations.

## Visual Abstract

**Figure:**
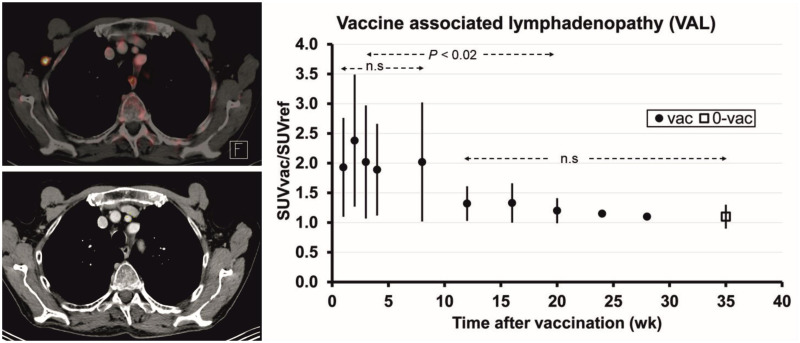

In these times of the global pandemic of coronavirus disease 2019 (COVID-19) infections and a rapidly increasing vaccinated population, vaccine-associated lymphadenopathy (VAL) in axillary or supraclavicular lymph nodes ipsilateral to the vaccination site on ^18^F-FDG examinations was increasingly observed (*1**–**6*). Ipsilateral axillary lymphadenopathy after intramuscular vaccine has been observed with seasonal and H1N1 influenza and human papilloma virus vaccines (*7**–**10*). These findings can impede interpretation of PET imaging, which poses an additional challenge for the workflow in nuclear medicine departments during the pandemic (*11**,**12*). The recognition of false-positive results is crucial to avoid unnecessary surgical reexploration or medical therapies. This has also been recognized by multidisciplinary recommendations of the scientific expert panel (*13*). However, to our best knowledge, there are only very limited data to which extent COVID-19 vaccinations induced increased uptake of prostate-specific membrane antigen (PSMA) ligands by regional lymph nodes (*5**,**14*). Given the recent approval of 2 PSMA imaging agents and the high incidence and prevalence of prostate cancer, the most common malignancy in men, it is very likely that many men will undergo PSMA PET imaging after a recent COVID-19 vaccination.

This study aimed to retrospectively assess the rate of VAL in PSMA ligand PET/CT and to investigate the characteristics and compare it with those of unvaccinated patients.

## MATERIALS AND METHODS

### Patient Population

Between June and July 2021, 265 consecutive patients with histologically confirmed intermediate- to high-risk prostate cancer who underwent ^18^F-rhPSMA-7.3 PET/CT, for staging, restaging, or planning of PSMA-targeted radionuclide therapy, were included in this retrospective study. In addition, the examinations of 9 additional advanced prostate cancer patients with known axillary lymph node metastases were analyzed to compare the features of axillary lymph node metastases with vaccination-related changes. In 3 of the 265 patients, clinical follow-up provided evidence for axillary lymph node metastases. These patients were subsequently excluded and were assigned and added to the group with known axillary lymph node metastases. All examinations were performed for clinical purposes as described by national and European guidelines (*11**,**15*).

In all patients, the date and the side of COVID-19 vaccination were recorded in the clinical notes. Informed written consent was obtained from all patients. The retrospective study was approved by the institutional review board (approval 719/21 S-NP).

### Synthesis of ^18^F-rhPSMA-7.3 and Administered Activity

^18^F-rhPSMA-7.3 was synthesized as described previously (*16*) under a license by the local authorities (Regierung von Oberbayern). Body weight–adapted activity with a median of 249 MBq of ^18^F‐rhPSMA-7.3 (mean, 258 ± 46; range, 174–400 MBq) was administered as an intravenous bolus with a median of 75 min (mean, 74 ± 11 min; range, 60–118 min) before examination.

### PET/CT Acquisition

All PET/CT examinations were performed on either a Biograph-Vision 600 or Biograph mCT scanner (Siemens Healthineers). All patients received diluted oral contrast (300 mg of ioxitalamate [Telebrix; Guerbet]) and 20 mg of furosemide. Diagnostic CT imaging was performed in the portal venous phase 80 s after intravenous injection of contrast agent (Imeron 300; Bracco Imaging) (1.5 mL/kg body weight; maximum, 120 mL) followed by the PET imaging in flow-mode. All PET examinations were acquired in 3-dimensional mode with an acquisition time of 1.1 mm/s. Emission data were corrected for randoms, dead time, scatter, and attenuation and were reconstructed iteratively by an ordered‐subsets expectation maximization algorithm (4 iterations, 8 subsets) including time-of-flight information and point spread function correction followed by a postreconstruction smoothing gaussian filter (5 mm in full width at half maximum). PET/CT images were reviewed (reviewers were dual–board-certified in radiology and nuclear medicine).

### Image Analysis

Axillary lymph node radiotracer SUV_max_, normalized for body weight, was measured by placing a region of interest at the axillary lymph node in the ipsilateral and contralateral side of the axillae. The corresponding CT images serve as orientation for the exact localization of the region of interest. Maximal diameter of the lymph node on CT was measured. The evaluating physician was masked for the site and time of the vaccination.

Because SUV_max_ depends on variable technical aspects, such as the use of different scanner, variable starting acquisition time after tracer injection, and patients’ conditions, such as clinical and oncologic status, the ratio between the SUV_max_ of the lymph node in the ipsilateral and contralateral reference sites (SUVratio) was calculated. Axillary lymph node uptake was defined as positive in the case of the ratio ≥ 1.5, as described previously by Thomassen et al. (*10*) for ^18^F-FDG and Eifer et al. (*5*). For unvaccinated patients, the SUVratio was calculated as a ratio between SUV_max_ of left to that of the right axillary lymph node.

### Statistical Analysis

Continuous variables were analyzed by descriptive statistics including arithmetic mean, SD, median, and range, whereas categoric variables were investigated by frequencies. Data of SUVratio were grouped according to the duration of the final vaccination in weeks and were plotted as a diagram. The Kruskal–Wallis test was used to compare SUVratio among patient groups with different time intervals between vaccine and PET examination. The Wilcoxon rank-sum test was used to compare SUVratio between patient groups with and without vaccine, as well as the pairwise comparison between patient groups in 2 different time intervals between vaccine and PET examination. The correlation between SUVratio and lymph node size as well as between lymph node size and elapsed time since the latest vaccination was analyzed by Spearman rank correlation. For all statistical analyses, statistical software SPSS (version, 25; IBM) was used. Significance level α was set at 0.05 with p < α.

## RESULTS

Two hundred thirty-three of 274 patients (85%) were vaccinated against COVID-19, either with mRNA or attenuated viral vectors–based approved vaccines, and 41 patients (15%) were not vaccinated. Two hundred sixty-two of 274 patients revealed no axillary lymph node metastasis, and the remaining 12 patients revealed axillary lymph node metastasis as confirmed by clinical data, lymph node exploration, or follow-up investigation.

Of the 233 vaccinated patients, 105 (45%) were noted to have increased PSMA ligand uptake in ipsilateral axillary lymph node compared with the contralateral site with positive SUVratio of ≥ 1.5.

**FIGURE 1.**
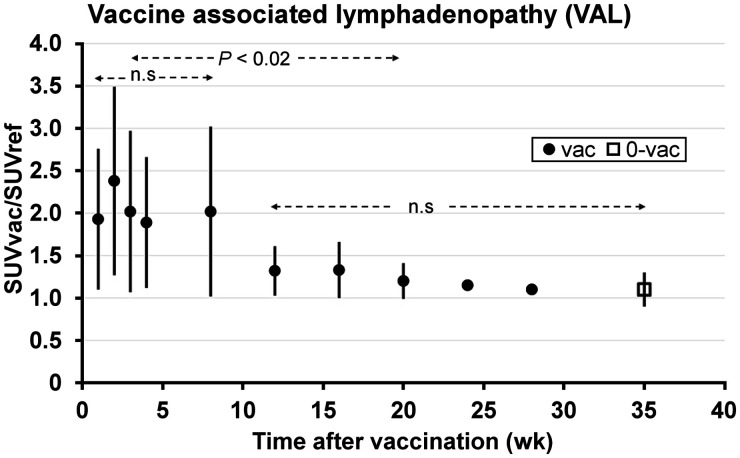
Temporal response of SUVratio after COVID-19 vaccination on PSMA PET/CT. Error bars indicate SD. vac = vaccinated; 0-vac = unvaccinated; n.s = statistically not significant.

The time course of PSMA ligand uptake in axillary lymph nodes after COVID-19 vaccination is shown in Figure 1. The temporal changes in lymph node SUVratio and size are listed in Table 1. Patients initially grouped in week 1–8 were regrouped in group A, respectively; patients initially grouped in week 12–28 were regrouped in group B. The comparison of SUVratio among the group in A and in B revealed no significant differences, whereas between A and B a significant decrease of SUVratio in B was registered (*P* < 0.02) and approached almost the level of unvaccinated patients. After 8 wk, the average lymph node size also dropped significantly (*P* < 0.01). The SUVratio and lymph node size in unvaccinated patients was 1.1 ± 0.2 (range, 0.6–1.5) and 11 ± 6 mm (range, 6–18 mm), respectively.

**TABLE 1.** Temporal Changes of Average Mean SUVratio and Average Mean Size of Axillary Lymph Nodes of Patients Without Axillary Lymph Node Metastasis and of Nonvaccinated Patients

Characteristic	Group		
	A	B	C
Time of vaccination	Between week 1 and week 8	Between week 8 and week 28	No vaccination
SUVratio			
Average mean	2.05	1.35	1.10
SD	±0.17	±0.09	±0.20
* P* value comparison of SUVratio between A and B → *P* < 0.02			
Size (mm)			
* *Average mean	23	19	11
* *SD	±10	±11	±6
* P* value comparison of lymph node size between A and B → *P* < 0.01			

Patients initially grouped in weeks 1, 2, 3, 4, and 8 were regrouped in A. Patients initially grouped in weeks 12, 16, 20, 24, and 28 were regrouped in B.

**FIGURE 2.**
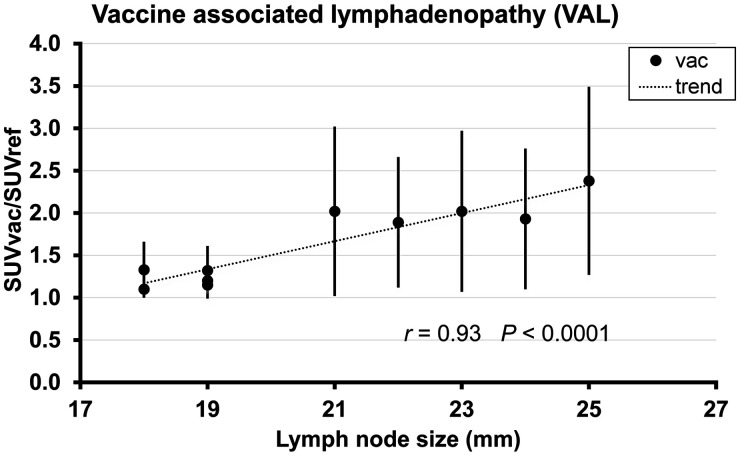
Relation between SUVratio and lymph node size on PSMA PET/CT. Error bars indicate SD. vac = vaccinated.

**FIGURE 3.**
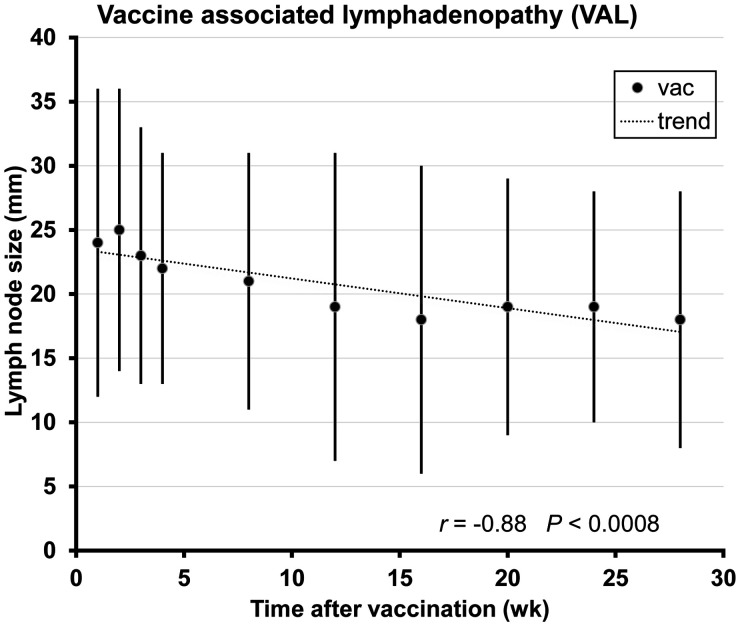
Relation between lymph node size and duration after vaccination. Error bars indicate SD. vac = vaccinated.

SUVratio and lymph node size were significantly correlated (*r* = 0.93 *P* < 0.0001, Fig. 2), and in the group of vaccinated patients there was a significant trend for the size of axillary lymph node to decrease over time after vaccination (*r* = −0.88 *P* < 0.0008, Fig. 3).

**FIGURE 4.**
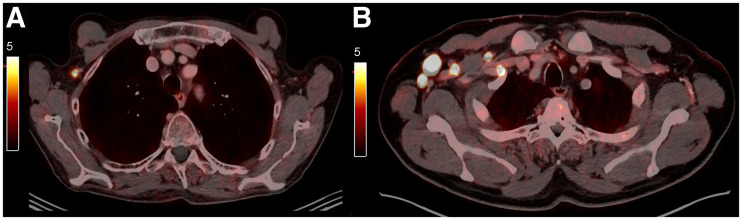
Representative PSMA PET/CT images of a patient with VAL (A) and of another patient with axillary lymph node metastasis (B).

Confirmed axillary lymph node metastases had a higher PSMA SUVratio of 14.92 ± 5.52 and were larger, with average size of 34 ± 12 mm, than nonmetastatic nodes (*P* < 0.001 and 0.001, respectively). In patients without axillary lymph node metastasis, the SUVratio and lymph node size after COVID-19 vaccination was 1.74 ± 0.87 (range, 0.3–4.7) and 22 ± 9 mm (range, 8–33 mm). Figure 4 represents an example of images of VAL in a patient without axillary lymph node metastasis and in another patient with axillary lymph node metastasis.

The subsequently confirmed metastatic axillary lymph nodes (*n* = 3) exhibited focal PSMA ligand uptake within groups of lymph nodes of variable size (8–33 mm). The SUVratio was 5.1 ± 1.35 (range, 4.0–6.6). These patients also had additional PSMA-expressing lesions in ribs and thoracic vertebrae.

## DISCUSSION

Increased ipsilateral axillary uptake of PSMA ligand is common and occurred in 45% of prostate cancer imaged at varying time intervals after COVID vaccination. This should be considered a source of false-positive findings in PSMA PET/CT. The increased PSMA ligand bindings appears to resolve 8 wk after vaccination. A recently published study in which VAL was assessed in a small subgroup of 31 patients with prostate cancer and PSMA ligand PET/CT showed similar findings of PSMA-avid lymph nodes after vaccination (*14*). The prevalence of VAL in our population was lower than in the previous study, presumably because we included a larger population with patients with a considerably longer period of time since vaccination. Eight weeks after the vaccination, axillary lymph node uptake was no longer significantly different from that in unvaccinated patients. The frequency of positive axillary lymph nodes on PSMA ligand PET/CT examinations in our series is comparable to recently published data for ^18^F-FDG PET/CT examinations after COVID-19 vaccinations (*1**–**5**,**13**,**17*). It is well established that activated lymphatic cells show increased metabolic activity and consequently accumulate more ^18^F-FDG. In contrast, the reasons for the increased uptake of the ^18^F-rhPSMA-7.3 are less clear. Contrary to its name, PSMA expression is not specific to prostate and PSMA is expressed in many other organs, including lymph nodes (*18**,**19*). We believe that in addition to the physiologic PSMA expression, COVID-19 vaccination–induced cellular and humoral immune responses in lymph nodes could augment the preexisting PSMA expression and thus enhance the avidity of PSMA ligands in nonmetastatic lymph nodes in patients with prostate cancer. Future research is necessary to determine whether uptake of small-molecule PSMA ligands by inflammatory lesions is due to off-target binding (e.g., to peptidases different from PSMA) or due to PSMA expression that has so far remained undetected by immunohistochemistry.

Furthermore, our results may impact clinical practice in the sense of precautions in scheduling, preparation, and interpretation of PSMA PET examination. There was significant overlap between PSMA ligand uptake in benign versus malignant axillary lymph nodes. Thus, intensity of tracer uptake cannot reliably distinguish between metastatic and inflammatory nodes after COVID-19 vaccination. Nevertheless, axillary lymph node uptake after COVID-19 vaccination alone should not be used as a criterion for tumor progression. Precautions similar to those recently published for ^18^F-FDG PET/CT examinations are probably reasonable for the interpretations of PSMA ligand PET/CT examinations after COVID-19 vaccinations (*13*).

The negative correlation between lymph node size and duration after vaccination indicates that statistically the average lymph node size tends to be smaller with increasing duration after vaccination. Our data are in concordance with the results reported previously on the basis of axillary-ultrasound imaging (*20*). The positive correlation between SUVratio and lymph node size indicates that statistically SUVratio tends to increase with increasing size of lymph nodes.

Our study had some limitations. Only patients vaccinated with FDA- (Food and Drug Administration) and EMA- (European Medicines Agency) approved COVID-19 vaccines (BioNTech-Pfizer, Moderna, Astra Zeneca, Johnson & Johnson) were included. Despite known differences in the immunologic response of the vaccines, separate analysis among those vaccines was not performed because this information had not been consistently reported in the clinical notes.

Since local clinical symptoms vary between different COVID-19 vaccines, future research is required to determine whether there are also differences between the vaccines with respect to PSMA ligand uptake.

We studied only patients imaged with ^18^F-rhPSMA-7.3, which is currently in late-stage clinical trials for prostate cancer imaging. The impact of COVID-19 vaccination on other PSMA ligands remains to be studied.

## CONCLUSION

PSMA-avid lymphadenopathy is common after COVID-19 vaccination. The time required after COVID-19 vaccination to allow for resolution of PSMA uptake of reactive axillary lymph node was 8 wk. During this period, care must be taken to avoid false-positive findings on PSMA ligand PET/CT examinations.

## DISCLOSURE

Matthias Eiber is named as an inventor on a patent application for ^18^F-rhPSMA-7.3 and reports research support from Blue Earth Diagnostics Ltd. and prior consulting activities for Blue Earth Diagnostic Ltd., Novartis, Telix, Progenics, Bayer, Point Biopharma, and Janssen. Wolfgang Weber has indicated that he is on advisory boards and receives compensation from Bayer, Blue Earth Diagnostics, Endocyte, Reflexion, Rayzebio, Vida Ventures, ITM, and Pentixapharm. He has received research support from Siemens, BMS, Ipsen, Imaginab, and Piramal. No other potential conflict of interest relevant to this article was reported.

## Notes

KEY POINTS **QUESTION:** What is the prevalence of VAL in PSMA PET? How long does VAL persist in PSMA PET? **PERTINENT FINDINGS:** The prevalence of VAL is 45% (in 105 of 233 vaccinated patients) and thus common. Temporary occurrence of VAL persists until 8 wk after last vaccination. **IMPLICATIONS FOR PATIENT CARE:** During 8 wk after COVID-19 vaccination care must be taken to avoid false-positive findings on PSMA PET/CT examinations.
